# ALKBH5-PYCR2 Positive Feedback Loop Promotes Proneural-Mesenchymal Transition Via Proline Synthesis In GBM

**DOI:** 10.7150/jca.84213

**Published:** 2023-05-29

**Authors:** Li Li, Mengting Yang, Xufeng Pu, Yu Tang, Fei Fei, Zhangzuo Li, Hanjin Hou, Qian Chen, Qiaowei Wang, Yuqing Wu, Ying Zhang, Caifang Ren, Aihua Gong

**Affiliations:** 1 Department of Cell Biology, School of Medicine, Jiangsu University, Zhenjiang, Jiang Su Province, China.; 2 Department of Pathology, School of Medicine, Jiangsu University, Zhenjiang, Jiang Su Province, China.; 3 Department of Medical Imaging, The Affiliated Hospital of Jiangsu University, Zhenjiang, Jiang Su Province, China.; 4 Department of Pathology, The Affiliated Hospital of Jiangsu University, Zhenjiang, Jiang Su Province, China.

**Keywords:** ALKBH5, PYCR2, proline, AMPK/mTOR, PMT.

## Abstract

AlkB homolog 5, RNA demethylase (ALKBH5) is abnormally highly expressed in glioblastoma multiforme (GBM) and is negatively correlated with overall survival in GBM patients. In this study, we found a new mechanism that ALKBH5 and pyrroline-5-carboxylate reductase 2 (PYCR2) formed a positive feedback loop involved in proline synthesis in GBM. ALKBH5 promoted PYCR2 expression and PYCR2-mediated proline synthesis; while PYCR2 promoted ALKBH5 expression through the AMPK/mTOR pathway in GBM cells. In addition, ALKBH5 and PYCR2 promoted GBM cell proliferation, migration, and invasion, as well as proneural-mesenchymal transition (PMT). Furthermore, proline rescued AMPK/mTOR activation and PMT after silencing PYCR2 expression. Our findings reveal an ALKBH5-PYCR2 axis linked to proline metabolism, which plays an important role in promoting PMT in GBM cells and may be a promising therapeutic pathway for GBM.

## Introduction

Glioblastoma multiforme (GBM) is the most prevalent type of primary intracranial tumor, accounting for 81% of all malignant brain tumors. Although rare, they have high mortality and morbidity 1. There are four subtypes of GBM: proneural (PN), neural (NL), classical (CL), and mesenchymal (MES) 2. Especially compared with the PN subtype, the MES subtype is more aggressive and immune-evasive, which is strongly associated with poor prognosis. The PN subtype can convert to the MES subtype during tumor progression, that is Proneural-mesenchymal transition (PMT), with Clinical WHO-IV representing the most aggressive form of glioma. Similar to epithelial-mesenchymal transition (EMT), PMT is a non-negligible factor in the progression and recurrence of GBM 3-5. According to the Chinese Glioma Genome Atlas (CGGA) database, the PMT score is strongly correlated with risky behavior and treatment resistance in GBM. However, indicators that drive PMT remain obscure in GBM.

ALKBH5 is an RNA demethylase and locates in nuclear to modulate N6-methyladenosine (m6A) modification to affect RNA metabolism 6. The role of ALKBH5 in cancer development varies depending on the tissue origin of cancer 6. ALKBH5 is upregulated in breast cancer, glioma, lung cancer, ovarian carcinoma, gastric cancer and colon cancer, and knockdown of ALKBH5 inhibits cancer cells viability, colony formation and migration 7-12. However, ALKBH5 is downregulated in bladder cancer, pancreatic cancer, and osteosarcoma, and ALKBH5 overexpression markedly reduces the proliferative, migratory, and invasive features of those cancer cells 13-15. In GBM, ALKBH5 is also upregulated and tightly associated with reduced patient survival 8. Previous evidence implies that silencing ALKBH5 suppresses the proliferation of glioblastoma stem-like cells 11, 16. ALKBH5 also promotes EMT in glioblastoma 17. Therefore, ALKBH5 is significantly correlated with the tumorigenesis of GBM 18. However, the role and mechanism of ALKBH5 in regulating PMT in GBM remains unclear.

Metabolic reprogramming has been proposed to be a hallmark of cancer. GBM cells also undergo metabolic reprogramming to provide nutritional support for rapid tumor growth to synthesize more nucleotides and lipids 19-21. Recent works have demonstrated that ALKBH5 regulate multiple pathways of glycose metabolism 18, 22-24. For example, overexpression of ALKBH5 promotes oxidative pentose phosphate pathway (PPP) flux by enhancing mRNA stability of glucose-6-phosphate dehydrogenase (G6PD) in gliomas 8. In addition to the glycolytic pathway, metabolic changes in cancer cells primarily involve amino acid metabolism. Inhibiting ALKBH5 upregulates the m6A levels of glutaminase 2 (GLS2) and inhibits the malignant biological behavior of gastric cancer cells 25. Besides, Glucose metabolism also affects amino acid availability 26. Thus, ALKBH5 shows the potential to directly or indirectly regulate amino acid metabolism.

In recent years, a large number of studies have demonstrated that proline metabolism plays an important role in metabolic reprogramming of cancer and increasing proline promotes the occurrence and progression of cancer 27. Pyrroline-5-carboxylate reductase 2 (PYCR2), a critical enzyme that catalyzes the final step in proline biosynthesis, converting pyrroline-5-carboxylate to proline, has recently been shown to be associated with several cancers 28, 29. PYCR2 is up-regulated in colon cancer and enhances cell proliferation, migration, and invasion 30. In addition, knockdown of PYCR2 inhibits the EMT of colorectal cancer cells 31. This is consistent with the trend that increasing proline or ALKBH5 overexpression promotes GBM or cancer progression. We speculate that there may be a synergistic regulatory relationship among ALKBH5, proline metabolism, and PYCR2. However, their specific roles, regulatory relationships and mechanisms in the development of GBM PMT remain unclear.

In this study, we show that ALKBH5 and PYCR2 are essential for GBM cell proliferation and migration. ALKBH5 can promote proline synthesis by upregulating PYCR2 expression. Furthermore, the ALKBH5-PYCR2 positive feedback loop promotes PMT in GBM. This suggests that ALKBH5 may provide a novel node for the treatment of GBM.

## Materials and Methods

### Clinical Database Analysis

The Chinese Glioma Genome Atlas (CGGA, http://www.cgga.org.cn) provided ALKBH5 and PYCR2 clinical data for 325 patients. The expression levels of ALKBH5 and PYCR2 were obtained from Gene Expression Profiling Interactive Analysis (GEPIA, http://gepia.cancer-pku.cn) and The Human Protein Atlas (HPA, https://www.proteinatlas.org). The GEPIA performs overall survival analysis based on ALKBH5 and PYCR2, and uses Log-rank test. Select a suitable expression threshold for splitting the high-expression and low-expression cohorts. And, cohorts thresholds can be adjusted that Cutoff-High(%) is defined as expressing samples above this threshold, and Cutoff-Low(%) is defined as expressing samples below this threshold. Detailed clinical parameters of all enrolled patients are available at the database web site or from the corresponding authors upon reasonable request.

### Cell culture

ATCC provides human glioma cell lines, including SW1783, U251MG, LN229, and U87MG cells (Manassas, VA, USA). At 37°C in a 5% CO_2_ incubator (Thermo Fisher Scientific, USA), cells were grown in high glucose Dulbeccos Modified EagleMedium (DMEM, Hyclone, Beijing, China) with 10% fetal bovine serum (Gibco, Carlsbad, CA).

### Quantitative Real-time qRT-PCR

Total RNA was isolated from human GBM cells using RNAiso Plus (Invitrogen, Carlsbad, CA) according to the manufacturer's recommendations. Real-time RT-PCR was conducted using the RevertAid First Strand cDNA Synthesis Kit (Vazyme, Nanjing, China) and the SYBR GREEN PCR Kit (Vazyme, Nanjing, China) following the manufacturer's instructions. The relative expression of the various genes was normalized to β-actin. The following sequences were used for the qRT-PCR primers, and the results were evaluated using the standard ΔΔCt method.

### Plasmid Construction and cell transfection

Our laboratory has previously generated the sh-EGFP, sh-ALKBH5, Flag-Vector, Flag-ALKBH5, and Flag-PYCR2 plasmids, and those were kept at the School of Basic Medical Sciences, Jiangsu University. The p3FLAGMyc-CMVTM14 expression vector and the pLKO.1-purowere purchased from Sigma-Aldrich. Sangon Biotech was the source for the siPYCR2 (Shanghai). 12 hours before transfection, SW1783 and U251MG cells were seeded at a density of roughly 60% in six-well plates. Each well combines 1 to 2.5 volumes of lipofectamine TM 2000 reagent (Invitrogen, Carlsbad, CA) with 1 to 2.5 volumes of Vector, Flag-ALKBH5, or Flag-PYCR2 plasmid. After 48 hours, RNAs were extracted for further experiments in the above cells. HEK293T cells were co-transfected with psPAx2 and pMD2.G plasmids with sh-EGFP, sh-ALKBH5, or sh-PYCR2 using lipofectamine TM 2000. Following transfection, the supernatants were collected 48 and 72 hours later. U87MG and LN229 cells were infected with 1 × 10^6^ recombinant lentivirus transduction units in the presence of 8 mg/mL polybrene (Sigma-Aldrich) and selected by puromycin (2 μg/mL) until all cells in the control group became nonviable.

### Western Blot Assay

The total protein of cells was extracted using a 2 × sodium dodecyl sulfate (SDS) loading buffer and was separated by 10% SDS polyacrylamide gel electrophoresis. Then, protein samples were transferred onto polyvinylidene fluoride membranes and blocked with a blocking solution. The membranes were incubated with primary antibodies overnight at 4°C followed by secondary antibodies for 1 h; membranes were washed with 1× tris-buffered saline with Tween 20 three times. Protein bands were visualized using chemiluminescence (Meilunbio, Dalian, China) and analyzed using ImageJ software. The primary antibodies included ALKBH5 (Proteintech, 16837-1-AP, 1:2000), PYCR2 (Proteintech, 17146-1-AP, 1:2000), E-cadherin (Proteintech, 20874-1-AP, 1:5000), N-cadherin (Proteintech, 22018-1-AP, 1:2000), Vimentin (Proteintech, 10366-1-AP, 1:2000), YKL-40 (Proteintech, 12036-1-AP, 1:500), mTOR (CST, 28983, 1:1000), p-mTOR (CST, 5536, 1:1000), AMPK (Proteintech, 10929-2-AP, 1:1000), p-AMPK (CST, 2531,1:1000), and β-tubulin (Thermo Fisher Scientific, MA5-11732, 1:2000).

### Cell Counting Kit-8 (CCK-8) Assay

In 96-well plates, 8 × 10^2^ transfected cells were seeded and grown at 37°C, 5% CO_2_ for six days. Each well received 10 μL of CCK8 solution (Vazyme, Nanjing, China), and cells were incubated at 37°C, 5% CO_2_ for 1 hour. Each well's absorbance was measured at 450 nm at the same time daily, and the results were analyzed using GraphPad Prism Version.

### Colony Formation Assay

A total of 1×10^3^ transfected cells were seeded in six-well plates and grown at 37°C, 5% CO_2_ for two weeks, while the medium was replaced every three days. The colonies were fixed with 4% paraformaldehyde for 30 minutes, stained for 30 minutes with 0.1% crystal violet, and then rinsed with phosphate-buffered saline (PBS; HyClone, Beijing, China), and the number of colonies were counted with a light microscope (MoticAE2000). In 96-well plates, 8×10^2^ transfected cells were seeded and grown at 37°C, 5% CO_2_ for six days. Each well received 10 μL of CCK8 solution (Vazyme, Nanjing, China) and 90 μL DMEM medium, and cells were incubated at 37°C, 5% CO_2_ for 1 hour. Each well's absorbance was measured at 450 nm at the same time daily, and the results were analyzed using GraphPad Prism Version.

### Transwell Migration and Invasion Assays

For the migration assay, glioma cells were transfected for 72 hours with the specified plasmids, trypsinized, and then resuspended in serum-free media. For the invasion assay, BD Matrigel basement membrane (BD Bioscience, Corning, NY) was applied to the top of the chamber; then, the migration assay was conducted. In the bottom chambers of both the migratory and invasion tests, 500 μL of DMEM containing 10% fetal bovine serum was added. The upper chamber was stained with crystal violet for 30 minutes after being dyed with 4% paraformaldehyde at 4°C for 30 minutes. The cells were subsequently examined using an inverted microscope.

### Wound Healing Assay

The 24-well plate containing transfected cells was seeded with 1×10^5^ cells per well. Upon reaching approximately 90% cell fusion, a 10 μL pipette tip scrape was made over the diameter. After changing to serum-free DMEM, a photograph of the selected area was taken. Photographs were taken 24 hours later at the same place to determine the relative distance of cell movement. The experiment was repeated three times, and the mean value was calculated.

### Proline Detection Assay

In glioma cells, the indicated plasmids were transfected. Collect the trypsin-digested glioma cells 72 hours later in a 15 mL centrifuge tube, centrifuge (800 rpm, 5 minutes), and discard the supernatant. Glioma cells were extracted using extraction buffer and ultrasonic cell disruption (power 20%, sonication 3 seconds, interval 10 seconds, repeated 30 times), followed by 10 minutes of sharking in a 100°C water bath and centrifugation at 10000g at room temperature. The proline level in the supernatant was determined using the Proline Content Assay Kit (Nanjing Jiancheng Bioengineering Institute, BCO295, 100T/96S), the absorbance of the sample was measured at 520 nm using an enzyme calibrator, and a standard curve was developed based on the concentration of the standard to determine the proline content.

### In-Vivo Study

The animal experiments were performed under standard conditions. Twenty-five nude mice (male, 4-6 weeks old) were used and purchased from the Animal Center at Jiangsu University. The mice were randomly divided into five groups with five mice in each group. At the same time, all of the mice were injected subcutaneously with 1×10^5^ cells transfected sh-EGFP plasmid, sh-ALKBH5 plasmid, or PYCR2-siRNA. We randomly chose two groups from three PYCR2-siRNA groups, when the average tumor volume was about 50 mm^3^, they were injected with a proline solution (5mm, 100µL) in the abdominal cavity, one of them also injected with 4mg/kg Rapamycin (Meilun, Dalian, China). Tumor growth was monitored every two days.

### Statistical Analysis

All data are displayed as the mean ± standard error of the mean, or simply standard error (SEM). Plots were generated using the student's t-test (two groups) or the one-way analysis of variance (ANOVA; multiple groups) and Prism 8.0 (GraphPad, San Diego, CA). P values less than 0.05 were considered statistically significant. Each experiment has been conducted a minimum of three times, and representative images are provided.

## Results

### ALKBH5 accelerates GBM cell proliferation, migration, and invasion

**Figure 1:**
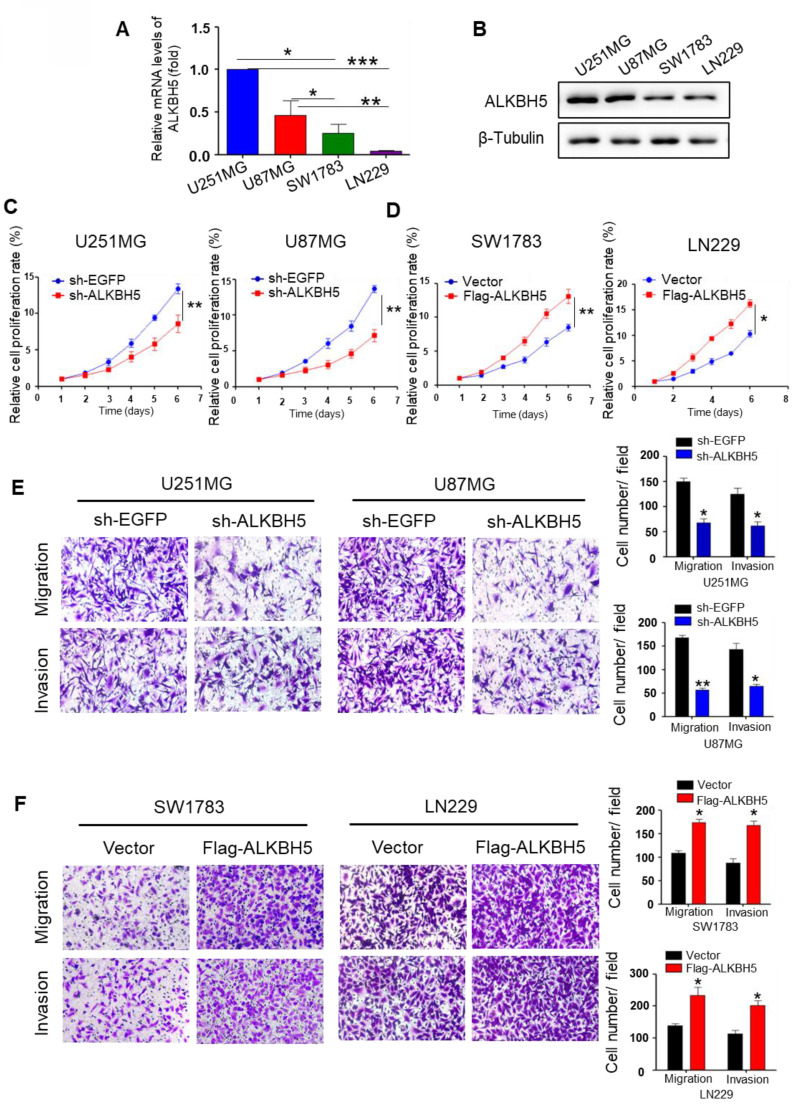
** ALKBH5 promotes GBM cell proliferation, migration, and invasion. (A)**Relative mRNA expression of ALKBH5 in U251MG, U87MG, SW1783, and LN229 cells (n=4). **(B)** Western blot for ALKBH5 protein expression in U251MG, U87MG, SW1783, and LN229 cells. **(C-D)** CCK-8 assay for cell proliferation in GBM cells transfected with sh-EGFP, sh-ALKBH5 or vector, Flag-ALKBH5. **(E-F)** Transwell assays for the migratory and invasive ability of GBM cells transfected with sh-EGFP, sh-ALKBH5 or vector, Flag-ALKBH5 plasmid, and the colony numbers were counted. Data are presented as the mean ± SEM. **P<0.01, ***P<0.001.

Real-time RT-PCR and western blot assays were performed to evaluate the baseline expression level of ALKBH5 in several glioma cell lines. The results demonstrated that U251MG and U87MG cells expressed higher level of ALKBH5 than SW1783 and LN229 cells (Fig. 1A-B). To further characterize the functional determinants of ALKBH5, we transfected the plasmids and confirmed their transfection efficiency using qRT-PCR and western blotting (Fig. S1A-D). CCK8 and colony formation data demonstrated that ALKBH5 knockdown inhibited the proliferation of U251MG and U87MG cells, whereas overexpression of ALKBH5 increased the proliferation of SW173 and LN229 cells (Fig. 1C-D, Fig. S1E). Transwell migration revealed that ALKBH5 silencing reduced migration of U251MG and U87MG cells (Fig. 1E). In contrast, overexpression of ALKBH5 stimulated the migration of SW1783 and LN229 cells (Fig. 1F), as well as the results of wound-healing tests (Fig. S1F-G). Then, we assessed cell invasion using the transwell BD Matrigel invasion test. The results demonstrated that silencing of ALKBH5 decreased the invasion of U251MG and U87MG cells relative to the control group (Fig. 1E-F). These results indicate that ALKBH5 promotes the proliferation, migration, and invasion of GBM cells.

### PYCR2 is indispensable for GBM cell proliferation, migration, and invasion

**Figure 2:**
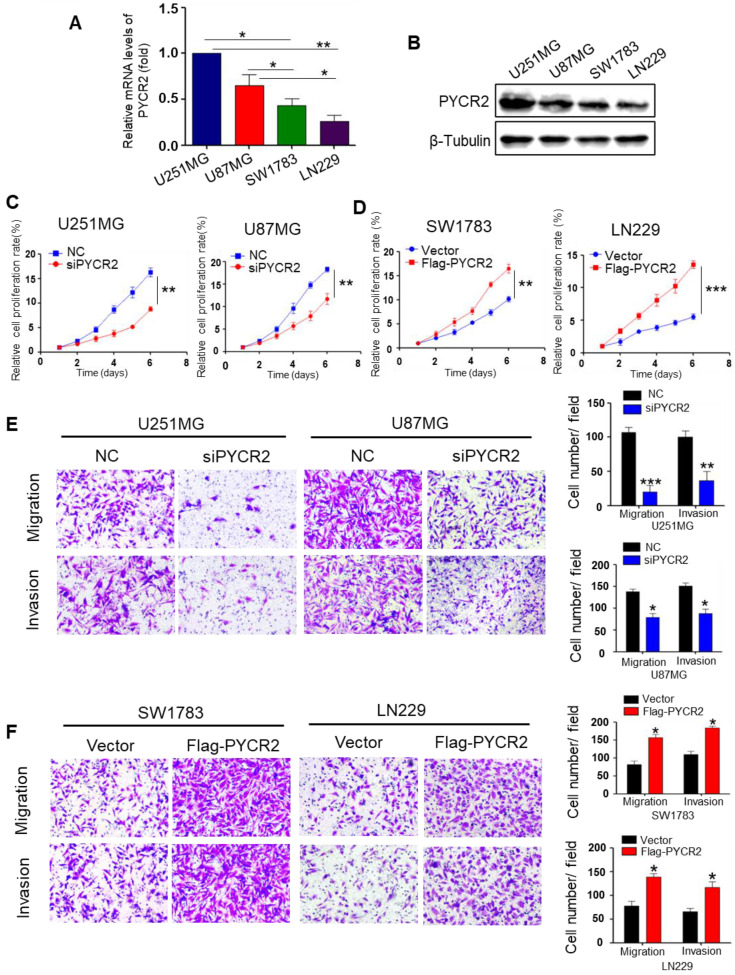
** PYCR2 enhances GBM cell proliferation, migration, and invasion. (A)** Relative mRNA expression of PYCR2 in U251MG, U87MG, SW1783, and LN229 cells (n=4). **(B)** Western blot for PYCR2 protein expression in U251MG, U87MG, SW1783, and LN229 cells.** (C-D)** CCK-8 assay for cell proliferation in GBM cells transfected with negative control (NC) siRNA or siPYCR2. **(E-F)** The effect of knockdown or overexpression of PYCR2 on migration and invasion rates in indicated GBM cells was detected by transwell assay. Data are presented as the mean ± SEM. *P<0.05, **P<0.01, ***P<0.001.

We first validated the basal expression of PYCR2 in various GBM cell lines. As shown in Fig. 2A-B, PYCR2 mRNA and protein levels were more abundant in U251MG and U87MG cells than in SW1783 and LN229 cells. Thus, PYCR2 was knocked down in U251MG and U87MG cells, and overexpressed in SW1783 and LN229 cells (Fig. S2A-D). The results of CCK-8 assay showed that silencing of PYCR2 decreased cell proliferation in U251MG and U87MG cells (Fig. 2C), while overexpression of PYCR2 promoted cell proliferation in SW1783 and LN229 cells (Fig. 2D). Using colony-forming assays, complementary data on the growth promotion of PYCR2 were also obtained (Fig. S2E). Using transwell migration and invasion experiment, we confirmed that PYCR2-siRNAs significantly reduced the motility and invasion of U251MG and U87MG cells (Fig. 2E), while PYCR2 overexpression in SW1783 and LN229 cells improved their motility and invasion (Fig. 2F). In addition, Wound-healing experiment confirmed that PYCR2 promoted GBM cell migration (Fig. S2F-G). These findings suggest that PYCR2 is a key gene for GBM cell proliferation, migration, and invasion.

### ALKBH5 and PYCR2 are abundantly expressed in GBM tissues and interact in GBM

**Figure 3:**
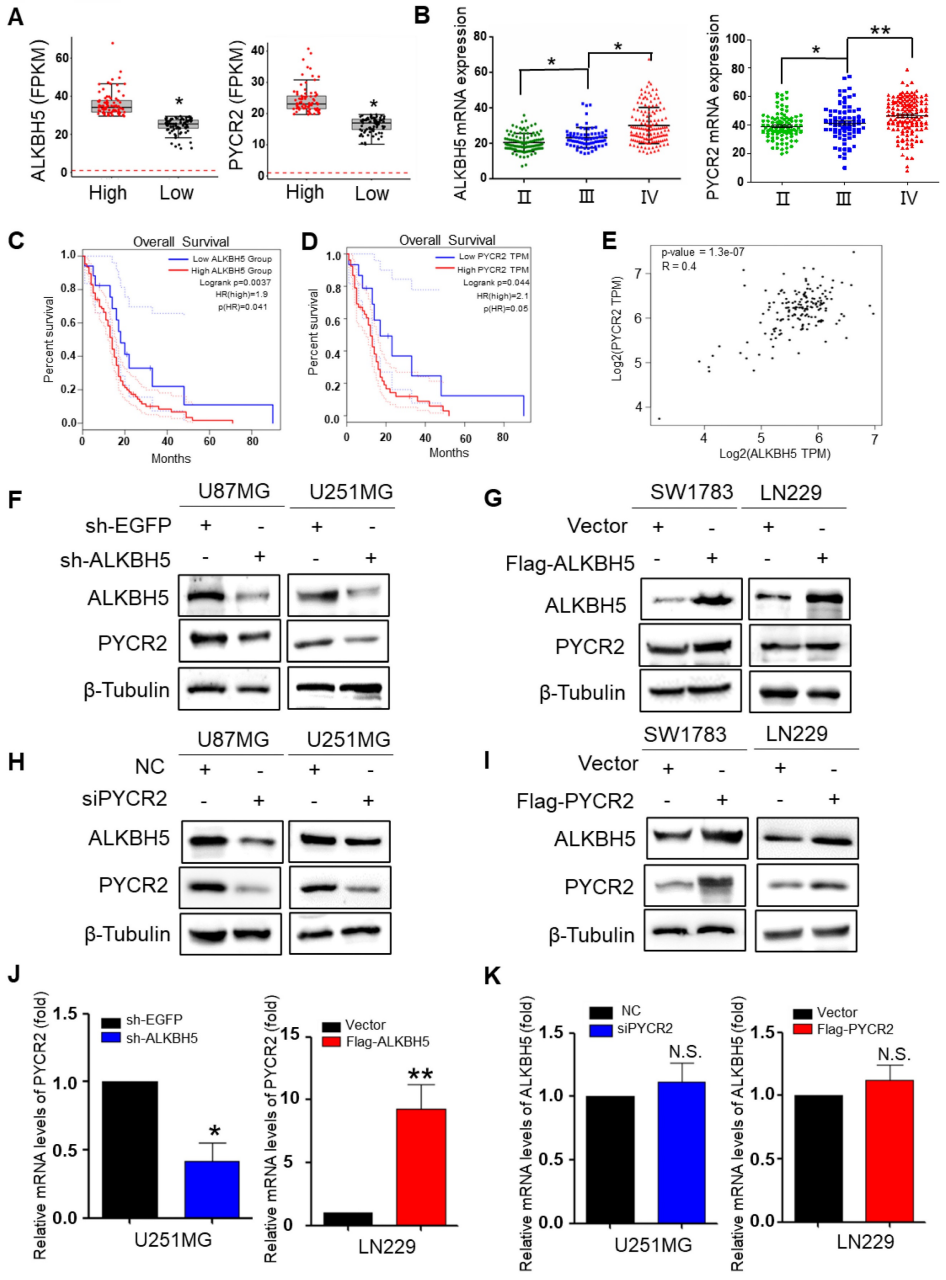
** ALKBH5 is correlation with PYCR2 in GBM cells. (A)** The boxplot of mRNA expression of ALKBH5 and PYCR2 in high-grade GBM and low-grade GBM (n=78). **(B)** Relative mRNA expression of ALKBH5 and PYCR2 in different grades of GBM (n=325). **(C)** The survival percentage of GBM patients with high (n=112) or low (n=17) ALKBH5 expression. **(D)** The survival percentage of GBM patients with high (n=54) or low (n=15) PYCR2 expression. **(E)** Correlation analysis of ALKBH5 and PYCR2 was conducted using the GEPIA database. **(F-G)** Western blot analysis for the indicated protein expression levels of PYCR2 when silencing or overexpressing ALKBH5 in GBM cells. **(H-I)** Western blot analysis for the protein expression levels of ALKBH5 after silencing or overexpressing PYCR2 in GBM cells. **(J)** qRT-PCR was used to detect the mRNA expression of PYCR2 after silencing or overexpressing ALKBH5 in GBM cells. **(K)** qRT-PCR was used to detect the mRNA expression of ALKBH5 after silencing or overexpressing PYCR2 in GBM cells. Data are presented as the mean ± SEM. *P<0.05, **P<0.01, N.S., not significant.

The Chinese Glioma Genome Atlas (CGGA) and The Human Protein Atlas (HPA) databases were utilized to investigate the relationship between ALKBH5 and PYCR2. The data demonstrated a statistically significant positive correlation between ALKBH5 and PYCR2. An integrated analysis of mRNA data of GBM tissues from 78 patients revealed that the expression of ALKBH5 in high-grade GBM was higher than that in low-grade GBM, and PYCR2 expression in GBM exhibited a consistent pattern with ALKBH5 expression (Fig. 3A). To plant the plots, we concentrated on the expression of ALKBH5 and PYCR2 in the CGGA database and evaluated all data. According to the WHO classification, ALKBH5 and PYCR2 were more highly expressed in grade IV glioblastoma (Fig. 3B). ALKBH5 and PYCR2 were also expressed at higher levels in high-grade GBM than in low-grade GBM, according to statistical analysis of the HPA database and pathological section (Fig. S3A-B). Survival studies also revealed that patients with low expression of ALKBH5 and PYCR2 had a better prognosis (Fig. 3C-D). In addition, the TCGA database revealed a medium positive connection between the expression of ALKBH5 and PYCR2 in GBM (Fig. 3E). These findings suggest that ALKBH5 and PYCR2 may be involved in regulating the malignancy of GBM. To further identify the positive relationship between ALKBH5 and PYCR2, ALKBH5 was silenced in U251MG and U87MG cells and overexpressed in SW1783 and LN229 cells. Western blot showed that ALKBH5 knockdown unexpectedly reduced the expression of PYCR2, while ALKBH5 overexpression boosted the expression of PYCR2 (Fig. 3F-G). The qRT-PCR results showed that PYCR2 expression is consistent with ALKBH5 expression in GBM cells (Fig. 3J and Fig. S3C). It means that ALKBH5 promotes PYCR2 expression maybe via modification of the mRNA. Knockdown of PYCR2 decreased the expression of ALKBH5, and overexpression of PYCR2 enhanced the expression of ALKBH5 in protein level (Fig. 3H-I). However, the mRNA expression of ALKBH5 had little change after silencing or overexpressing PYCR2 in GBM cells (Fig. 3K and Fig. S3D). Those results showed that PYCR2 promotes ALKBH5 expression happens mainly at the protein level.

### PYCR2 increases ALKBH5 expression via proline-AMPK-mTOR signaling

**Figure 4:**
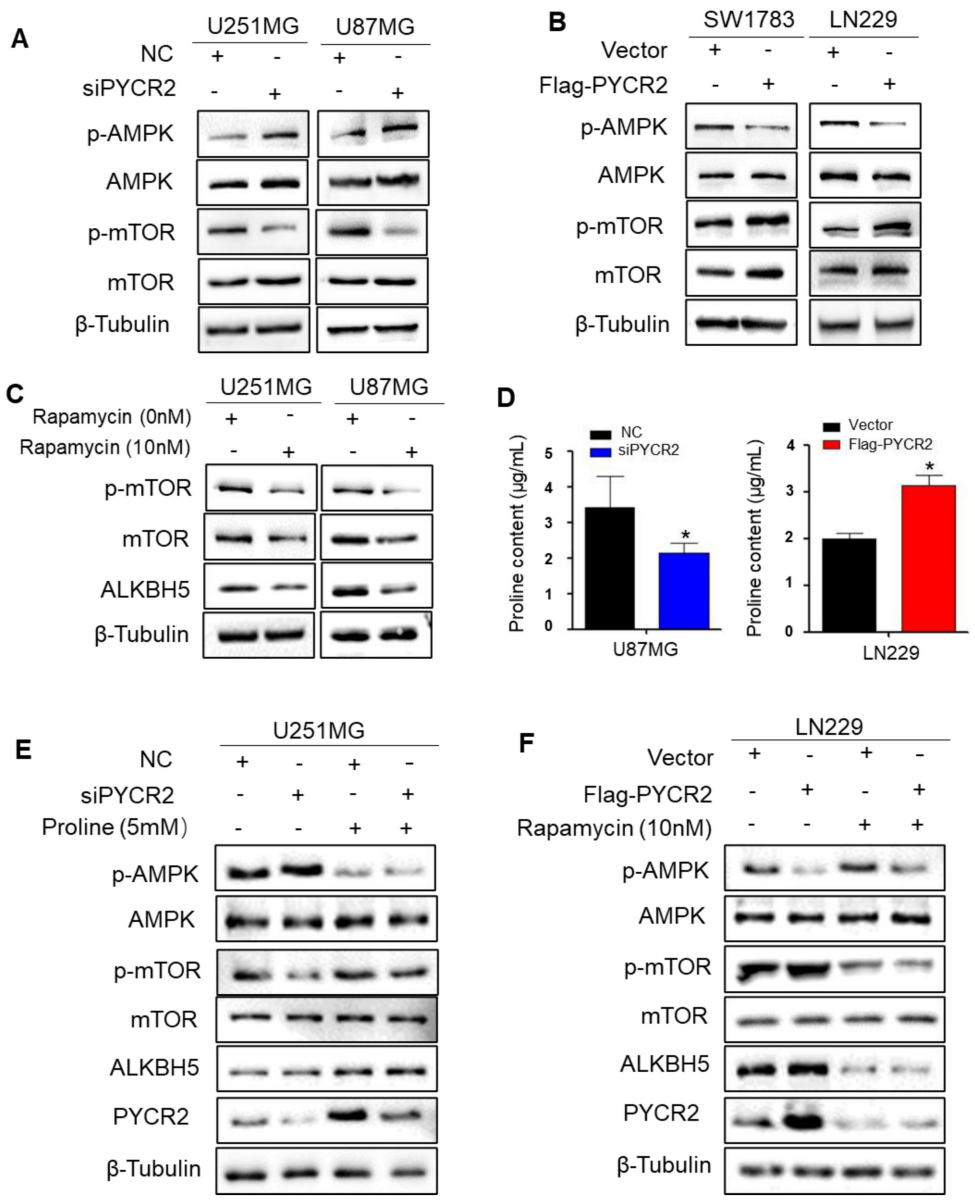
** PYCR2 upregulates ALKBH5 expression via proline-AMPK-mTOR signaling. (A-B)** Western blot analysis for the indicated protein expression levels of the AMPK, p-AMPK, mTOR, and p-mTOR when silencing or overexpressing PYCR2 in GBM cell©**(C)** Western blot analysis for the protein expression of ALKBH5 when inhibiting the mTOR in U251MG and U87MG cells. **(D)** Proline detection assay for measuring the proline content in silencing or overexpressing PYCR2 in GBM cells. **(E)** Western blot analysis for the indicated protein expression levels of the AMPK, p-AMPK, mTOR, and p-mTOR when silencing PYCR2 or adding proline in U251MG. **(F)** Western blot analysis for the indicated protein expression levels of the AMPK, p-AMPK, mTOR, and p-mTOR when overexpressing PYCR2 or adding rapamycin in LN229. Data are presented as the mean ± SEM. *P<0.05

The previous study confirmed a key role for AMPK-dependent regulation of proline metabolism in the energy homeostasis of the tumor microenvironment 32. The AMPK/mTOR pathway, a classical energy pathway, is involved in glycolysis as well as amino acid metabolism 33. PYCR2 indispensable for the regulation of energy metabolism, and studies have reported that PYCR2 silence activates AMPK/mTOR pathway induced autophagy in melanoma can regulate downstream genes through the AMPK/mTOR pathway 34. Then, we investigated whether AMPK/mTOR pathway is involved in the regulation of ALKBH5 by inhibiting or expressing PYCR2. Western blot results showed that knockdown PYCR2 dramatically increased the phosphorylation of AMPK (p-AMPK) and decreased the phosphorylation of mTOR (p-mTOR) (Fig. 4A). In contrast, p-AMPK expression dropped obviously while p-mTOR levels boosted after PYCR2 overexpression (Fig. 4B). Next, we explored the role of proline in the pathway of PYCR2 upregulates ALKBH5 expression by AMPK/mTOR signaling. The proline content was decreased after PYCR2 knockdown and increased after PYCR2 overexpression (Fig. 4D and Fig. S4A-B). Our results verified that proline not only upregulated ALKBH5 expression but also enhanced the p-AMPK expression and decreased p-mTOR expression (Fig. 4E). To further demonstrate that ALKBH5 is downstream of mTOR, GBM cells was exposed to rapamycin *in vitro*, a powerful and selective mTOR inhibitor. Western blot results demonstrated that mTOR inhibition greatly decreased the expression of ALKBH5, and proline failed to rescue ALKBH5 expression (Fig. 4F). These findings imply that PYCR2 promotes proline synthesis and then upregulates ALKBH5 expression via AMPK/mTOR signaling.

### ALKBH5-PYCR2 axis boosts PMT in GBM cells

**Figure 5:**
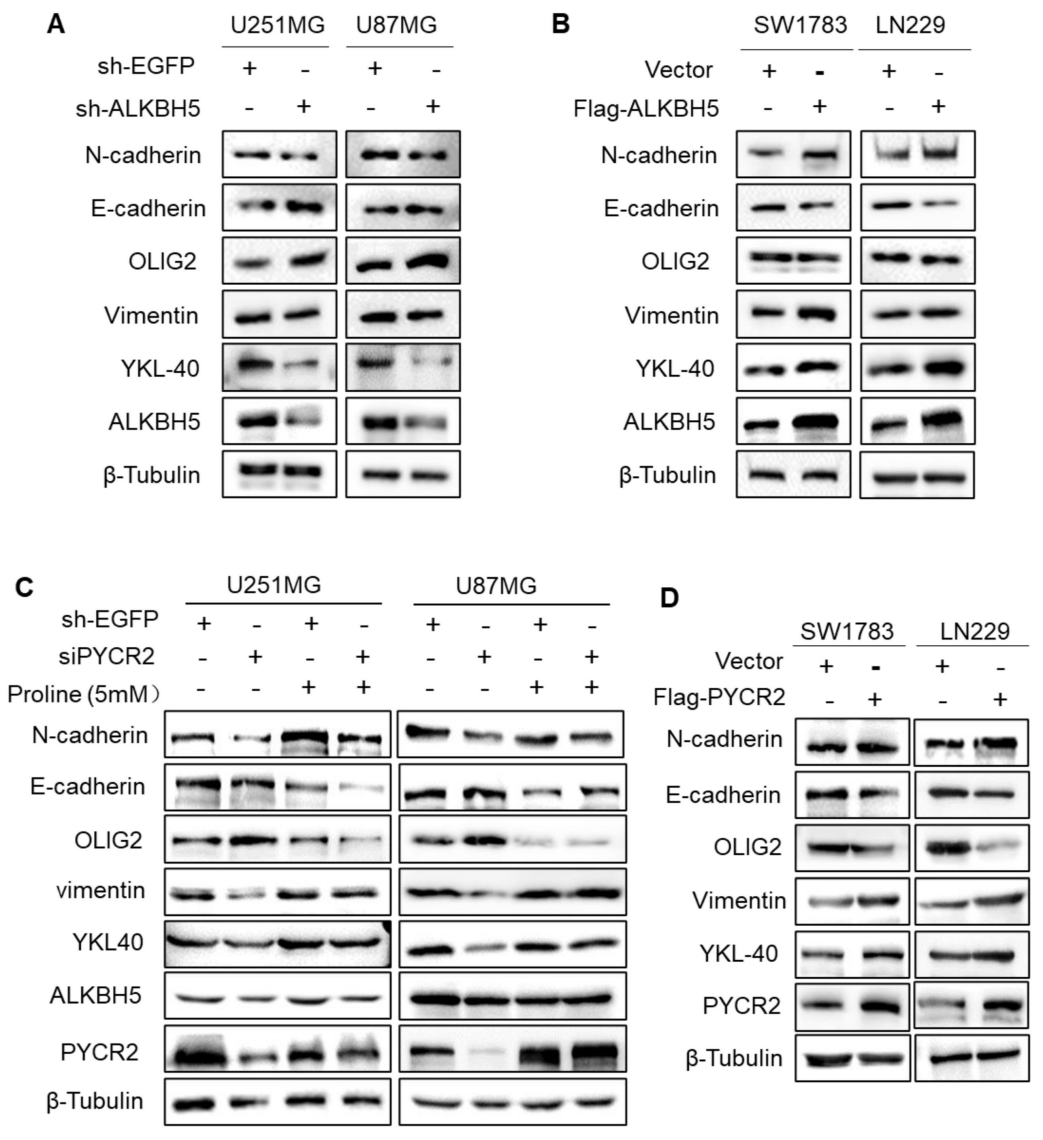
** ALKBH5-PYCR2 axis promotes PMT in GBM cells. (A-B)** The expression of PMT-associated proteins detected by western blot after silencing or overexpressing ALKBH5 in GBM cells. **(C)** Proline rescues PMT after silencing PYCR2 by detecting indicated proteins, and β-Tubulin served as the loading control. **(D)** The expression of PMT-associated proteins detected by western blot after overexpressing PYCR2 in GBM cells, and β-Tubulin served as the loading control. Data are presented as the mean ± SEM.

PMT was considered to be an early stage in the process of tumor migration 35. ALKBH5 knockdown decreased the expression of mesenchymal markers (N-cadherin, Vimentin, and YKL40) and elevated the expression of proneural marker OLIG2 in both U251MG and U87MG cells, as shown by Western blotting (Fig. 5A). And ALKBH5 overexpression in GBM cells efficiently enhanced the expression of PMT-related makers (Fig. 5B). As anticipated, overexpressing PYCR2 or adding proline promotes PMT, and the addition of proline to PYCR2-knockdown GBM cells partially reversed the occurrence of PMT (Fig. 5C-D). These findings indicate the biological function of ALKBH5-PYCR2-proline in GBM is promoting PMT, which may become one of the causes of the malignant progression of GBM.

### ALKBH5-PYCR2 axis is essential for supporting tumor growth *in vivo*

**Figure 6:**
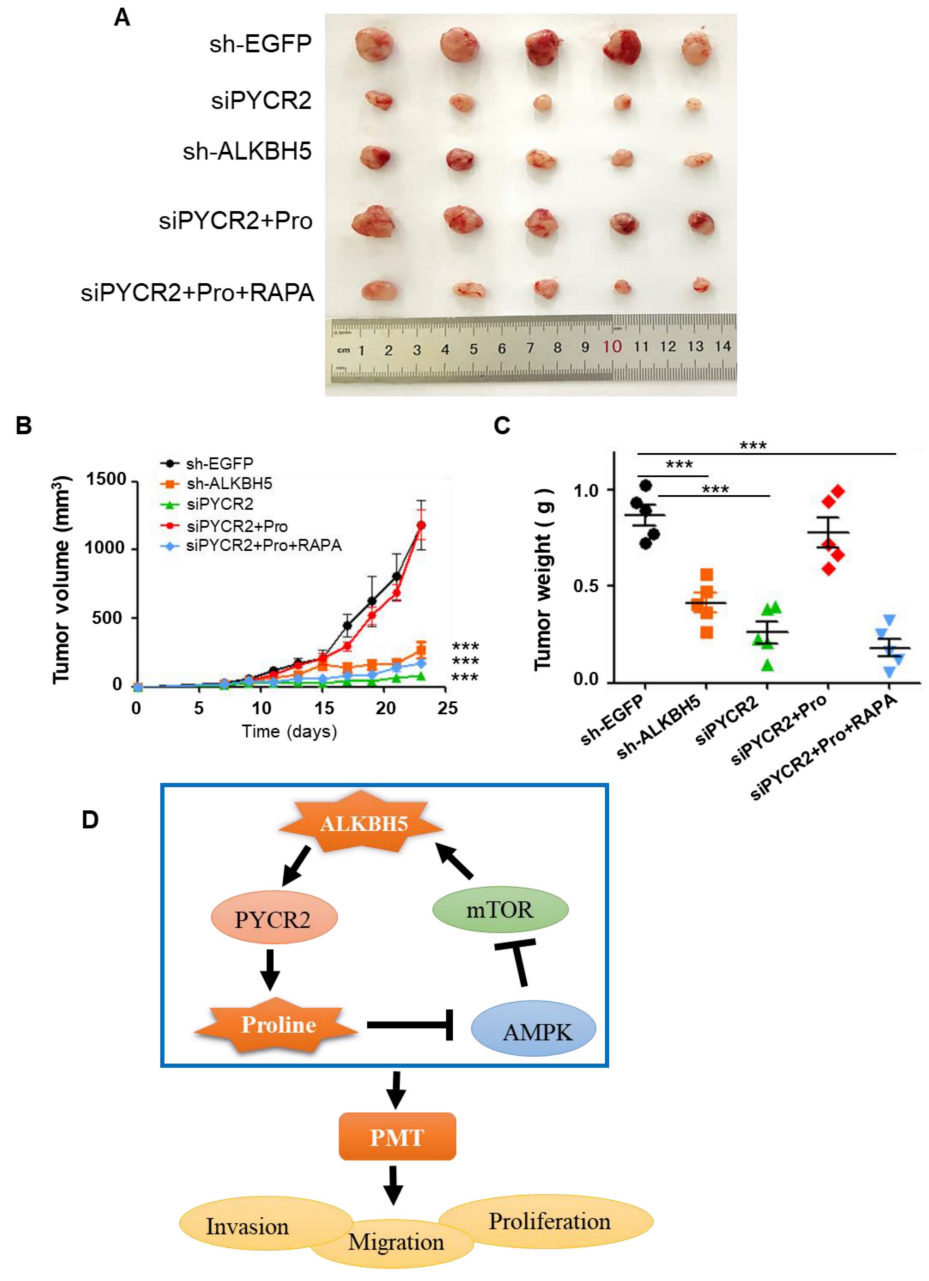
**ALKBH5-PYCR2 axis promotes tumor growth *in vivo.* (A)** Tumor volume change after different treatments. **(B)** Measurement of tumor volume size at different time points. **(C)** Tumor weight change per group on day 24. **(D)** Mechanistic diagram of the role of ALKBH5 in GBM. Pro: proline (5mM), RAPA: Rapamycin (10mM). Data are presented as the mean ± SEM. ***P<0.001

We further verified the function of ALKBH5-PYCR2 axis *in vivo*. The tumor volume and weight of the five groups have been shown in Fig. 6A-C. Both siPYCR2 and sh-ALKBH5 inhibited tumor growth. The addition of proline significantly promoted tumor growth; however, proline couldn't rescue tumor growth after inhibiting mTOR.

The above results show that, the ALKBH5-PYCR2 axis played an indispensable role in the malignant progression of GBM, and proline biosynthesis was probably one of the most important causes (Fig. 6D).

## Discussion

There is increasing evidence supporting the hypothesis that m6A modification and proline synthesis pathway are important in certain environmental conditions to modulate the tumor progression. Our data suggest that ALKBH5 promoted proliferation, migration, and invasion in GBM cells, which was consistent with previous findings 16. ALKBH5 has been shown to involve in the glycolytic pathways to alter the tumor microenvironment, however, few studies have shown its involvement in amino acid metabolism, particularly the reprogramming of proline metabolism 13, 22, 36. In this study, we found silencing ALKBH5 decreases proline content in GBM cells (Fig. S3B). In addition, our data demonstrated that ALKBH5 promoted proline biosynthesis primarily by upregulating PYCR2 at the mRNA level. Previous studies have shown that ALKBH5 stabilizes the targeted genes by lowering the m6A methylation 37, 38. In bladder cancer, ALKBH5 specifically recognizes m6A sites, reduces the stability of CK2α mRNA, and then alters the level of CK2α, thereby affecting the glucose metabolic pathway 13, 39. In glioblastoma, ALKBH5 promotes tumor growth by lowering the m6A methylation in target mRNA transcripts and increasing the FOXM1 expression 36. According to these evidences, it can be speculated that m6A demethylase ALKBH5 may promote PYCR2 expression by reducing m6A methylation, thereby ensuring the stability of PYCR2 mRNA thus promoting proline synthesis.

PYCR2 represents a critical enzyme for proline synthesis, and their regulation of GBM cell proliferation, migration and invasion has been demonstrated. This may be attributed to the regulation of energy production by proline metabolism during tumor growth 32. Previous studies have shown that proline indirectly regulates the well-known physiological energy sensor AMPK 40, 41. Our study further confirms that PYCR2 interference activates AMPK signaling. In addition, proline biosynthesis could divert glutamine carbons away from mitochondrial oxidation, thereby minimizing oxygen species (ROS) generation 40, 42. Increased ROS in the tumor microenvironment has been shown to activate AMPK and inhibit tumor initiation and progression 43. Thus, PYCR2 interference may directly regulate energy metabolism or increase ROS and disrupt redox homeostasis, thereby activating AMPK signaling and inhibiting tumor initiation and progression. This is consistent with the findings of this study that PYCR2 interference activates AMPK and inhibits GBM proliferation. The mTOR signaling is downstream of the AMPK signaling pathway and has long been verified to function at all stages of cell growth and coordinate environmental conditions 44. Activation of the AMPK/mTOR signaling significantly suppressed cell migration, invasion and proliferation in esophageal squamous cell carcinoma 45. In our study, knockdown of PYCR2 could increase the p-AMPK expression and decrease the p-mTOR expression, thus suppressing GBM cell proliferation, migration and invasion.

Intriguingly, we confirmed that PYCR2 promotes ALKBH5 expression and decreases mTOR phosphorylation. The exogenous addition of proline promoted ALKBH5 expression, while inhibition of mTOR resulted in no change of ALKBH5 compared to the control, which means ALKBH5 is controlled by mTOR (Fig. 5G). The p70S6 Kina se 1 (S6K1) and eIF4E Binding Protein (4EBP) as the downstream of mTOR signaling promoted mRNA translation and enhanced the spliced mRNA translation efficiency 46. We speculated that the mTOR signaling may promote the translation of ALKBH5 mRNA through certain protein kinases and increase the level of ALKBH5 protein, then affecting tumor metabolism. Although the molecular mechanism is unclear, this study demonstrates that PYCR2 enhanced the ALKBH5 expression via the AMPK/mTOR pathway. Thus, we first found that ALKBH5 and PYCR2 formed a positive feedback loop in GBM and altered the biological progression of GBM.

The PMT occurs in gliomas, similar to the EMT process, and is considered to be an important mechanism for promoting metastatic 35, 47, 48. The results of our study confirm that ALKBH5 contributes to formation of the MES phenotype and promotes PMT in GBM, which is consistent with the previous study that ALKBH5 can affect the malignant progression of GBM 49. In addition, we verified that proline synthesis promoted proliferation and PMT in GBM cells. This may be closely related to the fact that proline leads to collagen matrix formation and extracellular matrix (ECM) sclerosis (characteristic of human tumors), which promotes cancer cell survival, proliferation, and migration 32, 50. Although PYCR2 has been shown to promote EMT in colon cancer, we first confirmed that PYCR2 promotes PMT in GBM 31. The above evidences suggest that the ALKBH5-PYCR2 loop plays an important role in promoting the malignant progression of GBM *in vivo* and* in vitro.*

In summary, ALKBH5-PYCR2 in GBM forms a positive feedback loop and participates in promoting PMT. However, further work is needed to define the roles and mechanisms of ALKBH5 and PYCR2 in GBM, as our data and that of others clearly show that the network surrounding them is complex and remains poorly understood. This study on the ALKBH5-PYCR2 axis may provide a new strategy for preventing glioma progression.

## Supplementary Material

Supplementary figures.

## Figures and Tables

**Table 1:** Primers

Gene	Primers
*β-ACTIN*	Forward: 5′-CACCATTGGCAATGAGCGGTTC-3′;
	Reverse: 5′-AGGTCTTTGCGGATGTCCACGT-3′;
*ALKBH5*	Forward: 5′-CACATCCTGGAAGGCAGCAA-3′;
	Reverse: 5′-CCCCCAAAGTGGTGGTATCC-3′;
*PYCR2*	Forward: 5′-AGCTCCCCAGAAATGAACCTG-3′;
	Reverse: 5′-AGAGCCATGAATGCCTTCTCC-3′.

**Table 2:** Plasmid Oligonucleotides

Gene	Plasmid Oligonucleotides
Negative control	Sense: 5'-UUCUCCGAACGUGUCACGUTT-3'
	Antisense: 5'-ACGUGACACGUUCGGAGAATT-3'
PYCR2 siRNA	Sense: 5'-GCCCUUAAGACCCUCUUA-3'
	Antisense: 5'-UAAGAGGGUCUUCUUAAGGGC-3'
sh-EGFP	Sense: GATCCGTTCTCCGAACGTGTCACGTTTCAAGA GAACGTGACACGTTCGGAGAACTTTTTTG
	Antisense: AATTCAAAAAAGTTCTCCGAACGTGTCACGTTCTCTTGAAACGTGACACGTTCGGAGAACG
sh-ALKBH5	Sense: ATCCGAAAGGCTGTTGGCATCAATATTCAAGAGATATTGTGCCAACAGCCTTTCTTTTTTG
	Antisense: AATTCAAAAAAGAAAGGCTGTTGGCATCAATATCTCTTGAATATTGATGCCAACAGCCTTTCG
