# Supplementary material for: ALKBH5-PYCR2 Positive Feedback Loop Promotes Proneural-Mesenchymal Transition Via Proline Synthesis In GBM
Source: J Cancer. 2023 May 29;14(9):1579–91. doi: 10.7150/jca.84213 (PMC10266253; doi:10.7150/jca.84213)
Supplement: Supplementary file 1 — Supplementary figures. [file jcav14p1579s1.pdf]

Supplementary Data

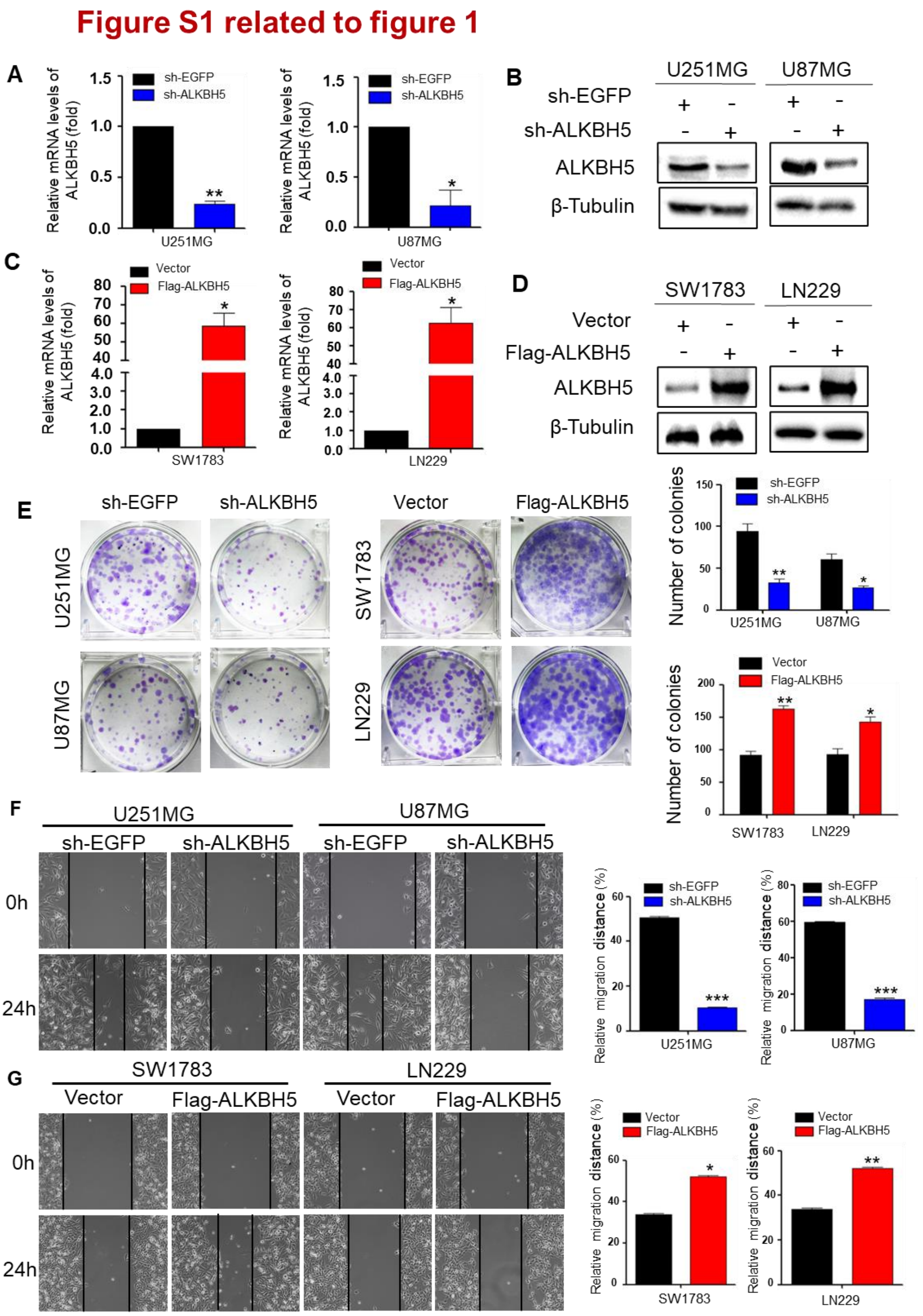

Fig. S1 (A-B) Relative mRNA and protein expression of ALKBH5 in U251MG and U87MG cells transfected with sh-EGFP or sh-ALKBH5 plasmid. (C-D) qRT-PCR and western blot for ALKBH5 expression in SW1783 and LN229 cells transfected with vector or Flag-ALKBH5 plasmid. (E) Colony-forming assay of cells transfected with

indicated plasmids in U251MG, U87MG (with sh-EGFP, sh-ALKBH5) and SW1783, LN229 (with vector, Flag-ALKBH5) cells, and the number of clones were counted. (F-G) GBM cells were transduced with overexpressing or silencing ALKBH5 plasmid and were determined migratory ability by wound healing assay. Data are presented as the mean ± SEM. *P<0.05, **P<0.01, ***P<0.001.

Figure S2 related to figure 2

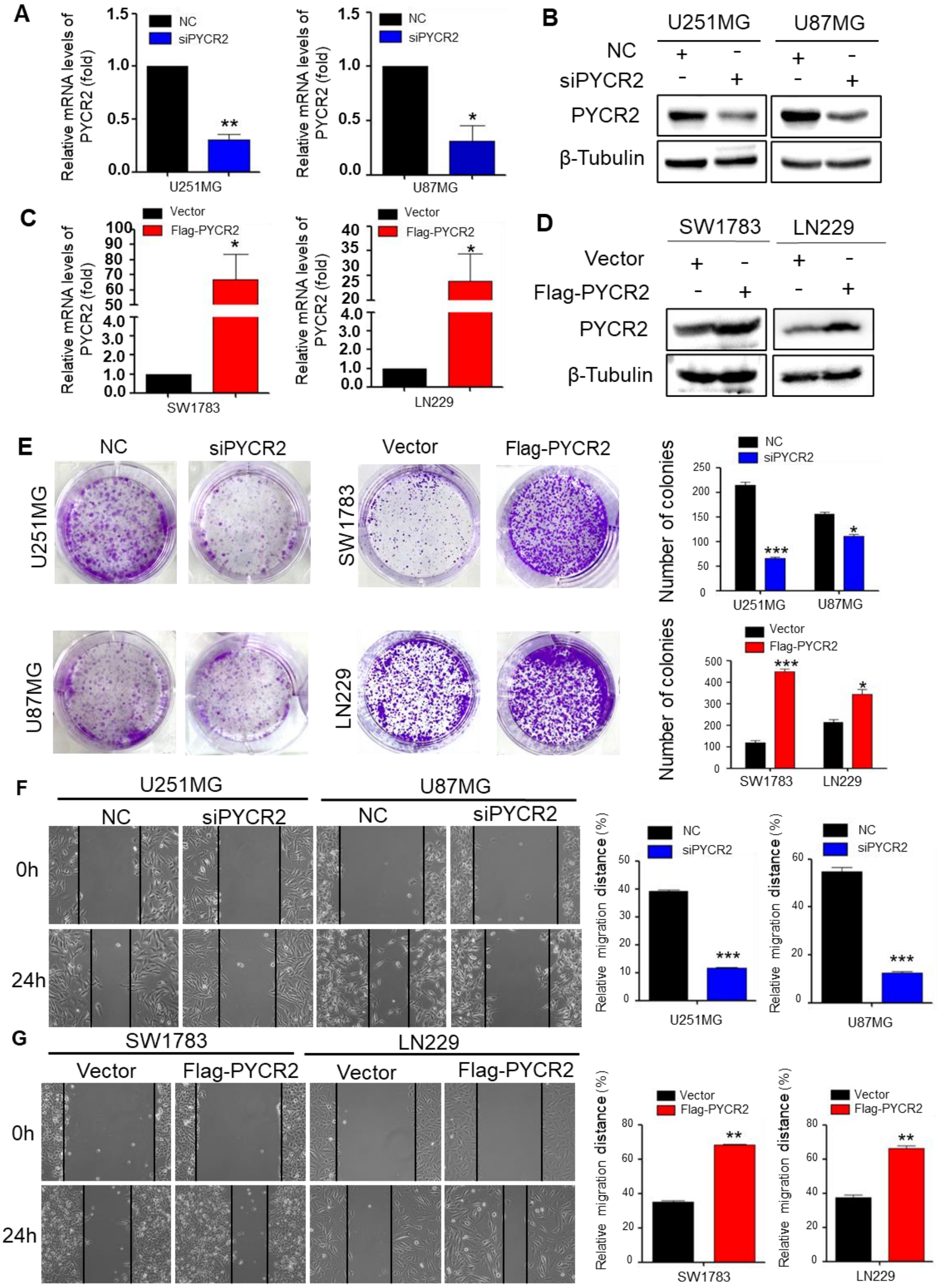

Fig. S2 (A-B) PYCR2 was depleted by siPYCR2 in the indicated cell lines. Cells were lysed for RT-PCR and Western blot analyses. (C-D) qRT-PCR and western blot for

PYCR2 expression in GBM cells transfected with vector or Flag-PYCR2 plasmid. (E) Colony-forming assay of cells transfected with indicated siRNAs or overexpression plasmids in various GBM cells and counted the number of clones. (F-G) The effect of knockdown or overexpression of PYCR2 on determining the migratory ability in indicated GBM cells was performed by wound healing assay. Data are presented as the mean ± SEM. *P<0.05, **P<0.01, ***P<0.001

Figure S3 related to figure 3

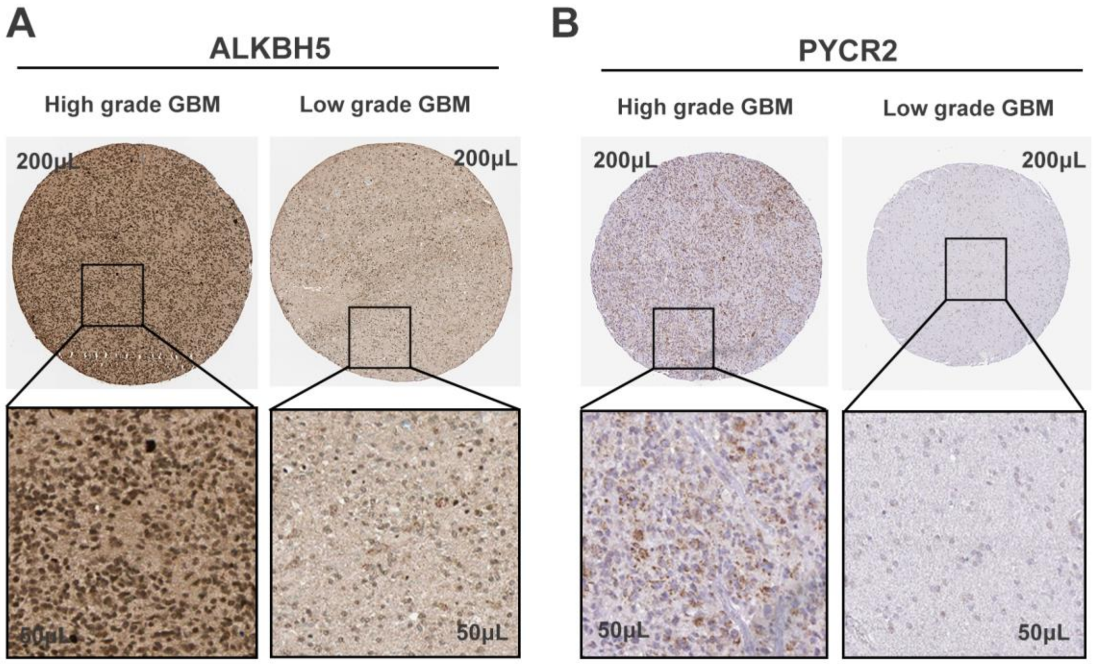

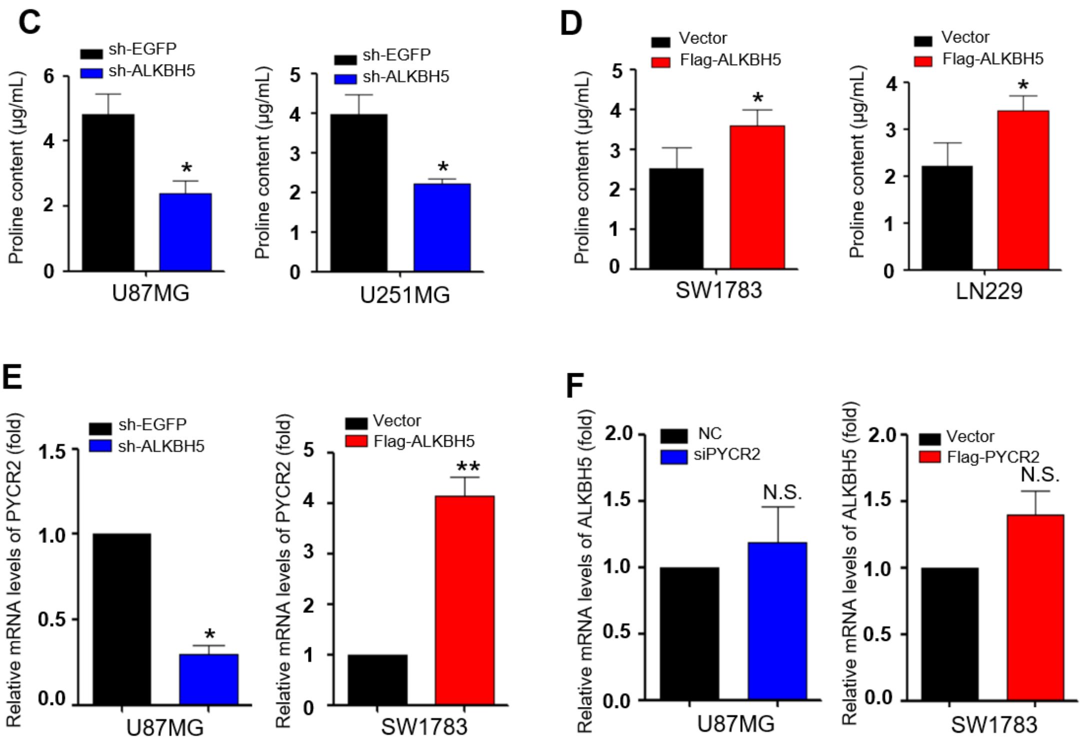

Fig. S3 (A) Immunohistochemical staining for ALKBH5 in high-grade GBM (patient id: 3241) and low-grade GBM (patient id: 3174). (B) Immunohistochemical staining for PYCR2 in high-grade GBM (patient id: 2908) and low-grade GBM (patient id: 2852). (C-D) Proline detection assay for measuring the proline content in silencing or overexpressing ALKBH5 in GBM cells. (E) qRT-PCR was used to detect the mRNA expression of PYCR2 after silencing or overexpressing ALKBH5 in GBM cells. (F) qRT-PCR was used to detect the mRNA expression of ALKBH5 after silencing or overexpressing PYCR2 in GBM cells. Data are presented as the mean ± SEM. **P*<0.05, ***P*<0.01, N.S., not significant.

Figure S4 related to figure 4

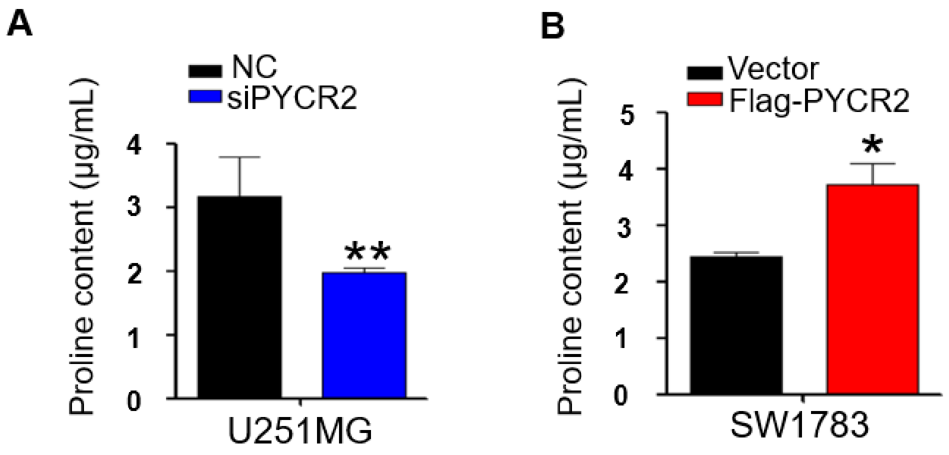

Fig. S4 (A-B) Proline detection assay for measuring the proline content in silencing or overexpressing PYCR2 in GBM cells. Data are presented as the mean ± SEM. *P<0.05, **P<0.01
